# Process Evaluation of the Tier 1 Program of the Project P.A.T.H.S.

**DOI:** 10.1100/tsw.2006.355

**Published:** 2006-11-16

**Authors:** Daniel T.L. Shek, Hing Keung Ma, Joyce H.Y. Lui, Daniel W.M. Lung

**Affiliations:** ^1^ Quality of Life Centre, Hong Kong Institute of Asia-Pacific Studies, The Chinese University of Hong Kong, Hong Kong, China; ^2^ Department of Education Studies, Hong Kong Baptist University, Hong Kong, China; ^3^ Social Welfare Practice and Research Centre, The Chinese University of Hong Kong, Hong Kong, China; ^4^ Social Welfare Practice and Research Centre, The Chinese University of Hong Kong, Hong Kong, China

**Keywords:** positive youth development, evaluation, Hong Kong

## Abstract

To understand the implementation quality of the Tier 1 Program of the Project P.A.T.H.S., two observers carried out process evaluation in six schools randomly selected from the participating schools in the form of systematic observations of 12 units. Results showed that the overall level of program adherence was generally high, ranging from 50% to 95%, with an average of 84.5%. High implementation quality of the program in the areas of student interest, student participation and involvement, classroom control, use of interactive delivery method, use of strategies to enhance student motivation, instructors familiarity with the students, opportunity for reflection, degree of achievement of the objectives, quality of preparation, overall implementation quality, and success of implementation was also observed. The findings provide support for the implementation quality of the program.

## INTRODUCTION

When a psychosocial intervention program is designed, one basic question is whether the developed program is effective. In the evaluation literature, many strategies have been proposed to evaluate the effectiveness of a psychosocial intervention program, such as objective outcome evaluation and subjective outcome evaluation. While the outcomes of a program are important to consider, it is equally important to appreciate the fact that the outcomes of an intervention program are contingent on the quality of program implementation. As such, it is crucial to understand the quality of the program implementation process.

According to Scheirer[1], process evaluation is "the use of empirical data to assess the delivery of programs …. Process evaluation verifies what the program is, and whether or not it is delivered as intended to the targeted recipients and in the intended dosage" (p. 40). Unfortunately, a survey of the literature shows that evaluation studies on adolescent prevention programs have based primarily on objective outcome evaluation. With reference to the public health literature, Linnan and Steckler[2]

commented that there is “a plethora of reports about interventions that have successful outcomes. A limited number of studies, however, disentangle the factors that ensure successful outcomes, characterize the failure to achieve success, or attempt to document the steps involved in achieving successful implementation of an intervention” (p. 1). In a review of over 1,200 published prevention studies, Durlak[3] showed that less than 5% of these studies reported findings on program implementation. In a meta-analysis of evaluation studies of primary and early secondary prevention programs published between 1980 and 1994, Dane and Schneider[4] showed that only 39 out of 162 evaluation studies documented procedures of fidelity. Domitrovich and Greenberg[5] also showed that among the 34 effective prevention programs under review, only 21% examined whether the effective intervention was related to outcomes.

Scheirer[1] stated that there are several reasons for conducting process evaluation. First, process evaluation can guard against Type III error (i.e., existence or nonexistence of program effect because of occurrence of activities different from those intended by the program developers). Second, feedback collected in the implementation process can promote fidelity in the implementation process. Third, process evaluation can help program developers to understand whether the intended targets receive the program. Fourth, process evaluation can help to identify factors that contribute to program success. Finally, program developers can use process evaluation findings to understand how the developed program can be implemented successfully in human organizations and communities that are always complex in nature.

A survey of the literature shows that there are many process variables related to the program outcomes. In a study of the factors associated with fidelity in substance use prevention curriculum guides, Ringwalt et al.[6] found that one-fifth of the workers implementing the program did not use the curriculum guide at all and only 15% of them followed very closely. Several factors were found to influence program fidelity, which in turn affected the effectiveness of the program adopted. These factors included opportunity for discretion in the coverage of program content, perceived effectiveness of previous prevention programs, perceived effectiveness of the program, support from school principal, and the nature of funding of the school (i.e., public vs. private school).

Nation et al.[7] pointed out that there are many factors that determine the success of an adolescent prevention program. Among these factors, process variables, such as varied teaching methods (i.e., use of a wide range of teaching methods that help the program participants to become aware of and understand problem behaviors and acquire the related psychosocial skills) and positive relationships with adults (e.g., worker), are important factors to be considered. There are research findings showing that teaching practices and program implementation attributes influence the extent of program success. To examine the hypothesized relationships between teaching practices and student behaviors in a comprehensive elementary school-based prevention program, Harachi et al.[8] reported findings supporting some of the propositions of the social development model that instructional strategies (proactive classroom management, cooperative learning methods, strategies to enhance student motivation, student involvement and participation, reading strategies, and interpersonal and problem-solving skills training) were related to student social competencies. Similarly, Tobler et al.[9] investigated what types of program were most effective in reducing, delaying, or preventing marijuana use and examined whether the characteristics of the participants and program implementation factors were related to program success. Results showed that programs with high peer interaction were more effective than programs with low peer interaction and that the delivery method instead of the program content determined the success of the program.

As the Project P.A.T.H.S. (**P**ositive **A**dolescent **T**raining through **H**olistic **S**ocial Programmes) is at its beginning stage in Hong Kong, it is important to consider its effectiveness. Based on the findings derived from the Experimental Implementation Phase, there are objective outcome evaluation findings[10] and qualitative evaluation findings[11] supporting the effectiveness of the Tier 1 Program. To further understand the program effectiveness, research findings based on a process evaluation study are presented in this paper. Besides adherence to the program (i.e., time and teaching materials specified in the curriculum manuals), the quality of implementation was also assessed in the study.

## METHODS

### Participants

Among the 52 schools joining the Experimental Implementation Phase, there were 29 schools adopting the full program (i.e., 20h program involving 40 units) and 23 schools adopting the 10h core program only. As it was desirable to observe the implementation process in schools adopting a less-intensive implementation mode where the program had been implemented for a sufficient period of time, relevant schools with implementation before January 2006 were invited to participate in the study. With reference to the above selection criteria, 12 schools adopting the full program and four schools adopting the core program constituted the sampling frame. Among these schools, five schools adopting the full program and one school adopting the core program were randomly selected to conduct the observations. The characteristics of the schools joining the process evaluation study can be seen in Table 1.

**TABLE 1**
**Summary of the Characteristics of the Schools Joining the Process Evaluation Study**

| School | A | B | C | D | E | F |
|---|---|---|---|---|---|---|
| **Background Characteristics of the Schools:** | | | | | | |
| Location (district) | Shatin | Kwai Chung | Tai Po | North Point | Yuen Long | Ma On Shan |
| Finance mode | Aided | Aided | Aided | Aided | Aided | Aided |
| Sex composition | Coeducational | Coeducational | Coeducational | Coeducational | Coeducational | Coeducational |
| Religious background | Nil | Nil | Christianity | Nil | Christianity | Nil |
| **Context of Observation:** | | | | | | |
| Choice of program | 20 h | 20 h | 10 h | 20 h | 20 h | 20 h |
| Mode | 15 sessions (1 h/session) and 2 sessions (2.5 h/session) | 40 sessions (30 mins/session) | 20 sessions (30 mins/session) | 20 sessions (1 h/session) | 20 sessions (1 h/session) | 20 sessions (1 h/session) |
| Integration with school curriculum | Liberal Studies | Civic Education and Extracurricular Activities | Class Teacher's Period | Civic Education, Life Education, and Project Learning | Liberal Studies | Life Education and Class Teacher's Period |
| No. of students in the class | 29 | 38 | 38-40 | 27 | 41 | 37 |
| Instructor(s) | Social workers and teachers | Social workers | Teachers | Social workers and teachers | Teachers and social workers | Teachers and social workers |
| Duration of observation | 66 mins | 68 mins | 90 mins | 88 mins | 78 mins | 68 mins |
| Unit observed | CC 1.1 and 1.2 | PI 1.2 and 1.4 | SE 1.4 and EC 1.1 | PI 1.3 and 1.4 | BF 1.1 and 1.2 | SE 1.1 and 1.2 |

### Procedures

For each school joining the process evaluation study, systematic observations of two teaching units were conducted. The units under observation covered five constructs, including self-efficacy, prosocial involvement, cognitive competence, emotional competence and beliefs in the future. The objectives of these units can be seen in Table 2. The observers were two research assistants of the project who were registered social workers. During the observations, each research assistant observed how the units were implemented and they were required to complete a rating form covering four major areas, including background information, integration with the curriculum, program adherence and fidelity, and quality of program delivery (see Appendix 1) in an independent manner. For program adherence and fidelity, the observers rated the degree of adherence and recorded the time used to implement the units. For the quality

**TABLE 2**
**Summary of the Objectives of the Observed Units**

| Schools | Units | Constructs | Objectives |
|---|---|---|---|
| A | CC 1.1 | Cognitive competence | To differentiate rational, creative, and critical thinking mentality. |
| | | | To understand the importance of reflection and the basic skills of reflection. |
| | CC 1.2 | Cognitive competence | To facilitate students to apply rational and critical thinking skills to analyze the problems of making friends via the Internet. |
| | | | To facilitate students to apply creative thinking in handling the problems of making friends via the Internet. |
| B | PI 1.2 | Prosocial involvement | To facilitate students to understand the definition of antisocial behavior. |
| | | | To facilitate students to differentiate prosocial behavior and antisocial behavior. |
| | PI 1.4 | Prosocial involvement | To facilitate students to understand what charity programs they can join in the community and the positive effects of it. |
| C | SE 1.4 | Self-efficacy | To master the goal-setting techniques of the “S.M.A.R.T.” strategy. |
| | | | To apply goal-setting techniques for setting a personal enhancement scheme. |
| | EC 1.1 | Emotional competence | To understand the basic concepts of emotion. |
| | | | To learn to describe different emotions with various vocabulary. |
| D | PI 1.3 | Prosocial involvement | To facilitate students to understand what charity programs they can join in the schools and the positive effects of it. |
| | PI 1.4 | Prosocial involvement | To facilitate students to understand what charity programs they can join in the community and the positive effects of it. |
| E | BF 1.1 | Beliefs in the future | To identify the pros and cons of optimism and pessimism. |
| | BF 1.2 | Beliefs in the future | To highlight the importance of beliefs towards the future. |
| | | | To facilitate the students to list out the things that students “can do”, ‘might do”, “able to do”, and “ought to do” in academic, family, and interpersonal aspects, etc. |
| F | SE 1.1 | Self-efficacy | To identify and assess self-efficacy in various aspects, such as academic domain, social life, appearance, and daily habit. |
| | SE 1.2 | Self-efficacy | To facilitate students to identify the influences of self-efficacy on personal feelings, thoughts, and behavior. |

of delivery, student interest, student participation and involvement, classroom control, use of interactive delivery method, use of strategies to enhance student motivation, use of positive and supportive feedbacks, instructors’ familiarity with the students, opportunity for reflection, degree of achievement of the objectives, time management, quality of preparation, overall implementation quality, and success of implementation were rated. The research assistants did not have any discussion and they were “blind” to the ratings of the partner when they completed the rating forms.

## RESULTS

For every unit, the ratings of each item by the two independent observers were averaged. To obtain an overall picture, the ratings for each item across all units were again averaged. The average overall adherence to the Curriculum Manuals was 84.5%, which was quite high (Table 3). For those units where modifications had been made, the observers regarded them as reasonable. As the ratings of the observers were averaged, it was necessary to know whether the ratings were reliable. Based on the overall adherence ratings across the 12 units, Pearson correlation analyses showed that the ratings across the two observers in the observed units (N = 12) were highly reliable (r = 0.81, $p < 0.01$).

**TABLE 3**
**Overall Ratings on Each Unit Observed in the Different Schools**

| **School** | **A** | | **B** | | **C** | | **D** | | **E** | | **F** | | **Average/Mean** |
|---|---|---|---|---|---|---|---|---|---|---|---|---|---|
| Unit observed | CC 1.1 | CC 1.2 | PI 1.2 | PI 1.4 | SE 1.4 | EC 1.1 | PI 1.3 | PI 1.4 | BF 1.1 | BF 1.2 | SE 1.1 | SE 1.2 | |
| Overall adherence (%) | 70 | 80 | 50 | 70 | 95 | 85 | 95 | 90 | 95 | 95 | 94 | 95 | 84.5 |
| **Ratings on a 7-point scale. The percentages of responses with ratings of 5 and above are in brackets.** | | | | | | | | | | | | | |
| 1. Student interest | 4.5 (50) | 5.5 (100) | 6 (100) | 4.5 (50) | 6.5 (100) | 6.5 (100) | 5.5 (100) | 4.5 (50) | 5 (100) | 5.5 (100) | 6 (100) | 7 (100) | 5.58 |
| 2. Student participation and involvement | 5 (100) | 6 (100) | 6 (100) | 5.5 (100) | 6.5 (100) | 7 (100) | 5.5 (100) | 5 (100) | 5 (100) | 5.5 (100) | 7 (100) | 7 100) | 5.92 |
| 3. Classroom control | 5.5 (100) | 5 (100) | 6 (100) | 5.5 (100) | 6.5 (100) | 6 (100) | 6 (100) | 5.5 (100) | 6 (100) | 5 (50) | 7 (100) | 6 (100) | 5.83 |
| 4. Interactive delivery method | 6.5 (100) | 6 (100) | 6.5 (100) | 4.5 (50) | 6.5 (100) | 6.5 (100) | 6 (100) | 4.5 (50) | 4.5 (50) | 5.5 (100) | 6 (100) | 7 (100) | 5.83 |
| 5. Strategies to enhance student motivation | 5 (100) | 5 (100) | 5.5 (50) | 4 (50) | 6 (100) | 6 (100) | 6 (100) | 5.5 (100) | 4 (50) | 5 (100) | 6 (100) | 6.5 (100) | 5.38 |
| 6. Use of positive and supportive feedbacks | 3.5 (0) | 3.5 (0) | 5.5 (100) | 3 (0) | 5 (100) | 5 (100) | 4 (50) | 3.5 (50) | 3.5 (0) | 4 (0) | 6 (100) | 6 (100) | 4.38 |
| 7. Instructors' familiarity with the students | 5 (100) | 5 (100) | 4 (0) | 4.5 (50) | 6.5 (100) | 6.5 (100) | 4 (50) | 4 (50) | 5.5 (100) | 5 (50) | 6.5 (100) | 6.5 (100) | 5.25 |
| 8. Opportunity for reflection | 6.5 (100) | 6.5 (100) | 4.5 (50) | 3 (0) | 5 (100) | 5.5 (100) | 5 (50) | 4 (0) | 4.5 (50) | 5.5 (100) | 5 (100) | 6 (100) | 5.08 |
| 9. Degree of achievement of the objectives | 6 (100) | 5.5 (100) | 5.5 (100) | 4 (50) | 6.5 (100) | 7 (100) | 6 (100) | 5.5 (100) | 5 (50) | 5.5 (100) | 6 (100) | 6.5 (100) | 5.75 |
| 10. Time management | 5.5 (50) | 5 (50) | 3 (0) | 2.5 (0) | 4.5 (100) | 4.5 (50) | 4.5 (50) | 4.5 (50) | 4.5 (50) | 6 (100) | 6 (100) | 6 (100) | 4.71 |
| 11. Lesson preparation | 5.5 (100) | 5.5 (100) | 6.5 (100) | 3 (0) | 6.5 (100) | 6.5 (100) | 5.5 (100) | 5.5 (100) | 5.5 (100) | 5.5 (100) | 7 (100) | 7 (100) | 5.79 |
| 12. Overall implementation quality | 6 (100) | 6 (100) | 6 (100) | 3.5 (0) | 6.5 (100) | 6.5 (100) | 5.5 (100) | 5 (100) | 5 (50) | 5.5 (100) | 6 (100) | 7 (100) | 5.71 |
| 13. Success of implementation | 6 (100) | 6 (100) | 5.5 (100) | 4.5 (50) | 6.5 (100) | 6.5 (100) | 6 (100) | 5.5 (100) | 5 (50) | 5.5 (100) | 6 (100) | 6 (100) | 5.75 |

Regarding the ratings for the quality of delivery, results in Table 3 revealed that the quality of implementation as assessed by the two observers was very high. An examination of the different areas showed that except the use of positive and supportive feedback and time management, the mean ratings were generally high. In particular, the implementation of the program was regarded as successful by the two observers. As the ratings of the observers were averaged, it was necessary to know whether the ratings were reliable. Based on the mean overall ratings across the 12 units, Pearson correlation analyses showed that the ratings across the two observers in the observed units (N = 12) were highly reliable (r = 0.80, $p < 0.01$).

## DISCUSSION

This paper attempts to examine program adherence and quality of implementation of the Tier 1 Program of the Project P.A.T.H.S. via systematic observations of 12 units delivered in six randomly selected schools. Two conclusions can be highlighted from the findings. First, with reference to the adherence of the program, results showed that the overall degree of adherence to the teaching units assessed by the two observers was on the high side. In addition, the two observers perceived that the objectives of the units implemented could be achieved (item 9 of Section D of the Appendix) and the overall quality of implementation was high (item 12 of Section D). These high ratings suggest that the fidelity of the program implementation was high. The second major conclusion of the study is that the different aspects of the program were perceived to be very positive. These aspects include (a) student interest and involvement (item 1 and item 2), (b) management and teaching strategies used by the instructors (items 3, 4, and 5), and (c) instructors' relationship with the students and effort (item 7 and item 11). Most important of all, the implementation was regarded as successful by the observers.

Nevertheless, there were three areas that deserve further attention. The first area is that the use of positive and supportive feedbacks in some of the units was not very high. The second area is the problem of time management. The findings suggest that the time management in some units was not desirable. The third area is that probably because of time constraint, opportunity for reflection was not high in some of the units. Obviously, these issues should be addressed in the refinement of programs and training provided to the instructors before they implement the program.

There are several limitations of the study. First, because of manpower constraints, only six schools were randomly selected to participate in this study. Although the number of schools participating in the study can be regarded as respectable, it would be desirable to include more schools with different characteristics to participate in the study. Second, besides the two research assistants, it would be helpful if more observers, particularly those unrelated to the project, can be involved in the observation and assessment process. Nevertheless, it should be mentioned that the inter-rater reliability of the independent and "blind" ratings of the two observers were on the high side. Third, besides adherence and the quality of implementation, process evaluation with reference to other dimensions, such as context of the implementation and the involvement of other stakeholders[12], would help the program developers to further understand the quality of the program implementation process. Finally, consistent with the intrinsic problem of all observation studies where time sampling is involved, one needs to be conscious of the degree of generalizability of the present findings to other temporal and spatial contexts. One possible confounding effect is that the students may become more cooperative when there are visitors and outside observers. Of course, the use of ethnographic strategies with prolonged engagement and observations would be helpful. Despite these limitations and in conjunction with the previous research findings[10,11], the existing research findings suggest that the quality of implementation of the Tier 1 Program was high and the program was helpful to the program participants.

## ACKNOWLEDGMENTS

The preparation for this paper and the Project P.A.T.H.S. were financially supported by the Hong Kong Jockey Club Charities Trust.

## APPENDIX 1

## P.A.T.H.S. TO ADULTHOOD: A JOCKEY CLUB YOUTH ENHANCEMENT SCHEME

## TIER 1 PROGRAM – OBSERVATION FORM

### A. Basic Information

(* Please copy this "☑" sign to the appropriate box.)

| | | | |
|---|---|---|---|
| Name of School: | | | |
| Form: ☐1 / ☐2 / ☐3 * Class: | | | |
| Number of Students: | Male: | Female: | |
| Number of Instructors: | Teacher(s): | Social Worker(s): | Others: |
| Sex of Instructors: | Male: | Female: | |
| Date of Observation: | | | |
| Duration of the Class Period: | | | |

### B. Integration with School's Formal Curriculum

(* Please copy this "☑" sign to the appropriate box.)

| |
|---|
| ☐ Incorporated into the formal curriculum (e.g. Life Education, Civic Education, Liberal Studies, etc.) Please specify the subject: ____________________<br>☐ Outside formal curriculum (e.g. after school, holiday, teachers' period etc.) Please specify:<br>☐ Others (Please specify: __________________________) |
| Is the instructor the Form Teacher of the Class?<br>☐ Yes ☐ No |

### C. Program Fidelity and Adherence

The Unit implemented:

Instructions:

1. Please fill in all the names of the activities and its expected duration in chronological order as specified in the curriculum manual.
2. Please tick ✓'none' if the activity was not carried out at all;
   please tick ✓ 'all' if the activity was carried out with strict or high degree of adherence to the planned curriculum;
   please tick ✓ 'part' if the activity was modified, and please specify the modifications, for instance: alteration of teaching strategies, omission of key points or role plays, discussions, etc.

| Activity | | Adherence to Planned Curriculum | | | Original Scheduled Time (mins) | Actual Time (mins) |
|---|---|---|---|---|---|---|
| | | None | Part (Estimated %) (specify modifications) | All | | |
| 1. | | | | | | |
| 2. | | | | | | |
| 3. | | | | | | |
| 4. | | | | | | |
| 5. | | | | | | |
| 6. | | | | | | |
| 7. | | | | | | |

Overall speaking, the estimated degree of adherence to the planned curriculum is _____%.

## D. Assessment of Curriculum Delivery

**1. STUDENT INTEREST**
How interested were the students in this unit?

| ☐ 1 | ☐ 2 | ☐ 3 | ☐ 4 | ☐ 5 | ☐ 6 | ☐ 7 |
|---|---|---|---|---|---|---|
| None or very few were interested | | | Half were interested | | | All or nearly all were interested |

**2. STUDENT PARTICIPATION AND INVOLVEMENT**
To what extent did the students participate in class activities?

| ☐ 1 | ☐ 2 | ☐ 3 | ☐ 4 | ☐ 5 | ☐ 6 | ☐ 7 |
|---|---|---|---|---|---|---|
| None or very few participated | | | Half participated | | | All or nearly all actively participated |

**3. CLASSROOM CONTROL**
To what extent was the class well controlled?

| ☐ 1 | ☐ 2 | ☐ 3 | ☐ 4 | ☐ 5 | ☐ 6 | ☐ 7 |
|---|---|---|---|---|---|---|
| Very poorly controlled | | | In between | | | Very well controlled |

**4. INTERACTIVE DELIVERY METHOD**
How interactive was the delivery method?

| ☐ 1 | ☐ 2 | ☐ 3 | ☐ 4 | ☐ 5 | ☐ 6 | ☐ 7 |
|---|---|---|---|---|---|---|
| Not interactive at all | | | Half interactive | | | Very interactive all the time |

**5. STRATEGIES TO ENHANCE STUDENT MOTIVATION**
To what extent were motivating strategies used to motivate the students?

| ☐ 1 | ☐ 2 | ☐ 3 | ☐ 4 | ☐ 5 | ☐ 6 | ☐ 7 |
|---|---|---|---|---|---|---|
| No motivating strategies at all | | | Half the time | | | Motivating strategies all the time |

**6. USE OF POSITIVE AND SUPPORTIVE FEEDBACKS**
How often were positive and supportive feedbacks elicited from the students?

| | | | | | | |
|---|---|---|---|---|---|---|
| □ | □ | □ | □ | □ | □ | □ |
| 1 | 2 | 3 | 4 | 5 | 6 | 7 |
| Not at all | | | Half the time | | | All or nearly all the time |

**7. INSTRUCTORS' FAMILIARITY WITH THE STUDENTS** (have to ask the instructors)
To what extent did the instructor know the students?

| | | | | | | |
|---|---|---|---|---|---|---|
| □ | □ | □ | □ | □ | □ | □ |
| 1 | 2 | 3 | 4 | 5 | 6 | 7 |
| Not at all | | | Average | | | Very well |

**8. OPPORTUNITY FOR REFLECTION**
To what extent was reflection encouraged?

| | | | | | | |
|---|---|---|---|---|---|---|
| □ | □ | □ | □ | □ | □ | □ |
| 1 | 2 | 3 | 4 | 5 | 6 | 7 |
| Not at all | | | Half the time | | | All or nearly all the time |

**9. EVALUATION OF THE DEGREE OF ACHIEVEMENT OF THE OBJECTIVES**
To what extent were the objectives achieved?

| | | | | | | |
|---|---|---|---|---|---|---|
| □ | □ | □ | □ | □ | □ | □ |
| 1 | 2 | 3 | 4 | 5 | 6 | 7 |
| Not achieved at all | | | In between | | | All or nearly achieved |

**10. TIME MANAGEMENT**
How well was the time managed?

| | | | | | | |
|---|---|---|---|---|---|---|
| □ | □ | □ | □ | □ | □ | □ |
| 1 | 2 | 3 | 4 | 5 | 6 | 7 |
| Very poorly managed | | | In between | | | Very well managed |

**11. LESSON PREPARATION**
How well was the lesson prepared?

| | | | | | | |
|---|---|---|---|---|---|---|
| □ | □ | □ | □ | □ | □ | □ |
| 1 | 2 | 3 | 4 | 5 | 6 | 7 |
| Poorly prepared | | | In between | | | Very well prepared |

**12. OVERALL IMPLEMENTATION QUALITY**
Overall, do you think the quality of implementation of this unit was high?

| | | | | | | |
|---|---|---|---|---|---|---|
| □ | □ | □ | □ | □ | □ | □ |
| 1 | 2 | 3 | 4 | 5 | 6 | 7 |
| Very low | | | Average | | | Very High |

**13. SUCCESS OF IMPLEMENTATION**
Overall, do you think the implementation of the program was successful?

| | | | | | | |
|---|---|---|---|---|---|---|
| □ | □ | □ | □ | □ | □ | □ |
| 1 | 2 | 3 | 4 | 5 | 6 | 7 |
| Very unsuccessful | | | Average | | | Very successful |
